# Adjunctive probiotic therapy enhances metronidazole efficacy in bacterial vaginitis: a clinical trial on Iranian women

**DOI:** 10.3205/dgkh000651

**Published:** 2026-06-02

**Authors:** Zahra Vahedpoor, Parisa Mamivand, Mohammad Javad Azadchehr, Mehdi Nazeri

**Affiliations:** 1 Autoimmune Diseases Research Center, Kashan University of Medical Sciences, Kashan, Iran; 2 Infectious Diseases Research Center, Kashan University of Medical Sciences, Kashan, Iran

**Keywords:** bacterial vaginosis, probiotics, metronidazole, recurrence, clinical trial, Lactobacillus plantarum, adjunctive therapy

## Abstract

**Background:**

Because probiotics have a positive effect on vaginal dysbiosis, it should be investigated whether the treatment of bacterial vaginosis (BV) with metronidazole can be improved by combining with a probiotic.

**Methods:**

The clinical trial enrolled married women aged 18–48 with BV and employed a rigorous double-blind, placebo-controlled design. The study compared the efficacy of a 7-day regimen of oral metronidazole (2×500 mg/d) and a vaginal probiotic (*Lactobacillus plantarum* 299v 1×10^9^ cfu/day over 21 days) to the same regimen with a placebo instead the probiotic.

**Results:**

The study enrolled 121 participants. Adding vaginal probiotics to metronidazole significantly reduced BV recurrence compared to metronidazole alone. With the combination therapy being well-tolerated and no serious adverse events reported

**Conclusions:**

The study suggest, that vaginal probiotics may be a promising adjunctive therapy for BV, although further studies are needed to confirm these findings and determine the optimal probiotic strains and dosage, as well as to investigate the potential benefits of probiotics for other aspects of BV management Other factors such as dietary habits, hygiene practices, and lifestyle were not controlled in this study and should be considered in future research.

## Zusammenfassung

**Hintergrund:**

Weil Probiotika die vaginale Dysbiose positiv beeinflussen, sollte untersucht werden, ob die Therapie der bakteriellen Vaginosis mit Metronidazol durch Kombination mit einem Probiotikum verbessert werden kann.

**Methode:**

In einer randomisierten, placebokontrollierten Doppelblindstudie wurden 121 verheiratete Frauen (18–48 Jahre) mit BV rekrutiert. Die Interventionsgruppe erhielt über 7 d orales Metronidazol (2×500 mg/d) plus ein vaginales Probiotikum (*Lactobacillus plantarum* 299v; 1×10^9^ KbE/d über 21 d). Die Kontrollgruppe erhielt Metronidazol plus Placebo. Primärer Endpunkt war die Rezidivprävention.

**Ergebnisse:**

Die Probiotika-Gruppe wies eine signifikant niedrigere Rezidivrate (9,7% vs. 37,3%; p≤0,05) sowie eine verbesserte Symptomremission bei Ausfluss, Dyspareunie und vaginaler Rötung auf (OR 3,75–5,12). Beide Therapien wurden gut vertragen.

**Schlussfolgerung:**

Die Studie legt nahe, dass vaginale Probiotika eine vielversprechende Zusatztherapie bei BV sein können. Allerdings sind weitere Studien erforderlich, um die Ergebnisse zu bestätigen, die optimalen Probiotikastämme und -dosierungen zu bestimmen sowie den potenziellen Nutzen von Probiotika für andere Aspekte der BV-Behandlung zu untersuchen.

## Introduction

Bacterial vaginosis (BV) is a prevalent vaginal infection associated with various adverse outcomes, including unpleasant symptoms, foul vaginal discharge, an increased risk of pelvic inflammatory disease, and preterm birth [1]. BV is caused by an imbalance in the vaginal microbiota and affects a substantial proportion of women in both developed (10–30%) and developing countries [2]. BV can occur in both pregnant and non-pregnant women and may manifest as vaginal discharge without inflammation [3]. Research in the past two decades has underscored the critical role of intestinal and urogenital microbiota in maintaining human health. Normal vaginal flora primarily consists of anaerobic bacteria, particularly *Lactobacillus(L.) crispatus* and *L. jensenii* [4]. However, BV disrupts this balance, leading to the predominance of bacteria such as *Gardnerella (G.)* spp., *Prevotella* spp., and *Atopobium* spp. Additionally, an increasing number of microorganisms, including Ba*cteroides fragilis, Peptostreptococcus, Mobiluncus, Sneathia, Leptotrichia, Propionibacterium, Fusobacterium, Veillonella* spp., *Mycoplasma hominis*, *Streptococcus* spp., *Staphylococcus* spp., *Bifidobacterium* spp., *Fusobacterium* spp., *Ureaplasma urealyticum*, and members of the Enterobacteriaceae family, have been identified in BV [5]. Evidence suggests that BV is associated with an increased risk of acquiring human immunodeficiency virus (HIV) and other sexually transmitted infections (STIs) [6]. Probiotics containing *Lactobacillus* spp. are often used and marketed for the management of BV and may be beneficial in preventing recurrent BV through recolonization. Efforts have been made to normalize vaginal flora by oral or vaginal administration of lactobacilli, which are believed to ascend to the vaginal tract after they are excreted from the body. Orally consumed probiotics are thought to ascend to the vaginal tract after excretion and may help in preventing BV recurrence. Therefore, daily consumption of probiotic products has been recommended to improve public health [7], [8]. 

The Amsel criteria are highly sensitive and specific tools for diagnosing BV. A positive diagnosis requires at least three out of four criteria: thin, homogeneous vaginal discharge, vaginal pH >4.5, a positive whiff test, and the presence of clue cells on microscopic examination. However, the use of the Amsel criteria necessitates a vaginal swab for discharge, microscopy, and slide/wet preparation with potassium hydroxide (KOH) solution. Culture-based diagnostic techniques are not useful in diagnosing BV due to the presence of anaerobes and *G. vaginalis* in vaginal secretions. The mainstay of treatment for BV is antibiotics, which have been shown to be effective [9]. However, antibiotic use can disrupt the balance of the vaginal microbiota, leading to adverse outcomes such as a reduction in Lactobacillus spp. and increased growth of other microorganisms, which can result in recurrent infection and treatment failures. In recent years, there has been a growing interest in probiotics, which are live microorganisms that confer health benefits when consumed in adequate quantities [10]. Research and market growth in probiotics as well as clinical trials involving probiotics and infectious diseases are expanding. Advances in BV management have prompted research into the potential of probiotics to improve treatment outcomes and maintain the vaginal bacterial flora. One proposed approach involves combining oral metronidazole with probiotics. If this intervention is effective in preventing BV recurrence, probiotics could complement antibiotic treatment as an adjunctive therapeutic measure for BV infection [11]. Other factors such as dietary habits, hygiene practices, and lifestyle were not controlled in this study and should be considered in future research. 

## Method

### Trial design and participant

This clinical trial, conducted at the Imam Reza Gynecology Clinic affiliated with the Kashan University of Medical Sciences, was meticulously registered on Iranian Registry of Clinical Trials (IRCTID: IRCT20180612040071N2) and approved by the Institutional Ethics Committee (No. IR.KAUMS.MEDNT.REC.1398.067). This study employed a rigorous double-blind, placebo-controlled design, the trial spanned from May 2020 to Jun 2023. Patient eligibility was determined based on stringent criteria for BV, as delineated in the 2021 guidelines for the management of sexually transmitted diseases [12]. The inclusion criteria adhered to the Amsel criteria, necessitating the presence of at least three out of four key symptoms or signs: gray vaginal discharge, elevated vaginal pH exceeding 4.5, detection of clue cells, and a positive whiff test result [13]. The study exclusively enrolled married women aged between 18 and 48 years. Conversely, the exclusion criteria were recent antibiotic usage (either systemic or intravaginal) within the preceding two weeks, immunocompromised or HIV-positive individuals, women with concurrent vaginal candidiasis, menopausal women, and pregnant or breastfeeding women. 

The patients were randomly selected from admission to the gynecology clinics of Imam Reza Hospital and were subsequently assigned to one of the two distinct groups. One cohort (n=62) was administered a 7-day regimen of 500 mg oral metronidazole twice daily. Additionally, this group received a vaginal probiotic tablet (Lacto Flora Fem) containing *L. plantarum* 299v, with a concentration of 1×10^9^ colony-forming units per day, administered for 72 h. This probiotic tablet was ingested once daily over a 21-day period,. The control group (n=59), was administered the same oral metronidazole regimen, in terms of dosage and duration. Furthermore, they were administered a vaginal probiotic tablet. Notably, both placebo and probiotic tablets were indistinguishable in their physical attributes, including color, shape, size, and packaging. The tablets were procured from Tasnim Pharmaceutical Company in Tehran, Iran. After completing their respective treatments, all patients were re-evaluated approximately 30–35 days later using the Amsel criteria and Nugent score, whichres ranged from 4 to 6, reflecting the presence of clue cells, or scores exceeding 7 without the presence of clue cells. 

Participation in the study was elective. Informed written consent was optioned from all participants prior to the start of interviews. Consent included the use of participant data and interview transcripts for the purpose of publication. All procedures performed in this study were in accordance with the ethical standards of the institutional and national research committees and align with the World Medical Association (WMA) Helsinki declaration, such as the ICH-GCP guidelines and CIOMS international ethical standards. 

### Clinical and bacterial assessment

Patients, who presented with a constellation of typical vaginitis symptoms, including unusual vaginal discharge, itching, burning, and dyspareunia, underwent a comprehensive evaluation conducted by a gynecologist. Vaginal secretions were collected using two sterile swabs to assess bacterial presence and measure vaginal pH levels. 

### Sample size determination

A post-hoc power analysis was conducted to assess the adequacy of the sample size in detecting clinically significant differences. The sample size was determined based on data from a prior investigation [14]. Notably, those who consumed probiotics displayed a significantly higher rate of recovery than those who received placebo (87.5% versus 67.5%). Each group comprised 64 participants with a 95% confidence interval and 80% power.

### Randomization and blinding

The randomization sequence was created by the software Stat Trek application, which generated random numbers independently by employing a robust and validated methodology. Additionally, the allocation and randomization procedures were concealed from the investigators, with an impartial supervisor overseeing the entire process to safeguard the objectivity and impartiality of the study’s outcomes.

### Outcomes

The primary outcomes encompassed a range of symptoms such as vaginal discharge, vaginal pruritus, dyspareunia, dysuria, vaginal inflammation marked by erythema, and abdominal discomfort. Clinical recovery was defined as the complete absence of all Amsel criteria or the presence of just one criterion in combination with Nugent scores falling within the ranges of 0–3 or 4–6, provided there were no clue cells detected.

### Statistical analysis

Results were analyzed and compared using the chi-squared test and Fisher’s exact test. All statistical analyses and results were performed and extracted using the SPSS software version 25. A statistical significance level of p=0.05 with a 95% confidence interval was used to determine statistical significance.

## Results

Although 121 participants were initially enrolled, seven individuals (two from the intervention group and five from the control group) withdrew from the trial. The reasons for withdrawal included loss to follow-up (n=5) and personal reasons unrelated to the study treatment (n=2). No adverse events were observed among the participants with bacterial vaginosis who received probiotic supplementation. The mean age of the patients in intervention and control group were 35.92±7.01 and 34.71±8.01 years respectivly. Baseline demographic characteristics, including the number of children, sexual activity, duration of infection, and contraceptive methods, showed no significant differences between the two groups (P>0.05) (Table 1 (Tab. 1)). 

The most commonly reported complaints in both treatment groups were primarily vaginal discharge, followed by abdominal discomfort, vaginal odor, painful sexual intercourse, and itching. Conversely, dysuria was the least frequently cited concern in both groups. Following treatment, most vaginal symptoms demonstrated substantial improvement in both groups. The intervention group exhibited a significantly greater degree of improvement across several symptoms, including vaginal discharge, dyspareunia, and vaginal odor (see Table 2 (Tab. 2) for detailed P-values). The exception was dysuria, for which the difference between groups did not reach statistical significance (P>0.05).

According to the Nugent scoring system, BV with a score ranging from 7 to 10 or from 4 to 6, alongside the presence of clue cells after treatment, was observed in 9.7% of the probiotic group compared to 37.3% of the placebo group. A significant difference between the two groups following treatment is shown in Table 2 (Tab. 2).

The group receiving probiotic supplementation exhibited statistically significant results (P=0.05) across several symptoms, including burning, discharge, dyspareunia, vaginal redness, vaginal odor, and abdominal pain, determined by logistic regression analysis. The odds ratios for these symptoms were 3.25, 3.75, 4.36, 5.12, 8.86, and 3.06, respectively. However, no significant differences (P>0.05) were observed between the two groups regarding itching and vaginal pH ≥4.5. Notably, there was a marked distinction in improvement assessed by the Nugent score, with the probiotic supplementation group having a favorable odds ratio of 3.06 (Table 2 (Tab. 2)).

Following a 35-day follow-up period, both groups exhibited clinical and bacteriological enhancements among patients who initially met the Amsel criteria. These criteria included a negative Whiff test, absence of homogeneous discharge, absence of clue cells, and vaginal pH <4.5. Although the probiotic-treated group displayed more pronounced improvement, there were no significant differences in clinical efficacy based on the Amsel criteria between the two groups. Nevertheless, a significant disparity in bacteriological improvement was evident, with 64.3% in the placebo group and 83.3% in the probiotic group, illustrating a notable difference between the groups (P≤0.05) (Table 3 (Tab. 3)). 

## Discussion

BV is the most common vaginal infection in reproductive-aged women, with a global prevalence of 10% to 30%. It is characterized by a shift in the vaginal microbiota from a predominance of *Lactobacillus* spp. to a diverse community of anaerobic and *Gardnerella* spp.. BV is associated with a number of adverse outcomes, including increased risk of pelvic inflammatory disease, preterm birth, and sexually transmitted infections [15].

Recent research has shown interest in using additional probiotic therapy to improve the effectiveness of metronidazole in treating BV [5]. The study revealed that 96% of the women in the probiotic group and 53% in the placebo group experienced complete recovery from BV, indicating a significant enhancement in metronidazole’s efficacy when combined with probiotics [16], [17].

The use of probiotics as an adjunctive treatment for bacterial vaginosis (BV) has been supported by a growing body of evidence. Probiotics are live microorganisms that can help maintain the balance of the vaginal microbiota and reduce the risk of BV recurrence [18]. A meta-analysis of randomized controlled trials found that probiotics were effective in reducing BV recurrences, and that the combination of metronidazole and probiotics was more effective than metronidazole alone in treating BV [19]. However, not all studies have found a significant benefit of probiotics as an adjunctive treatment for BV. One study found that oral probiotic adjunctive treatment did not increase the cure rate of BV patients compared to metronidazole alone. More strong evidence is needed to confirm the effectiveness and safety of probiotics in treating BV [1]. Other factors such as dietary habits, hygiene practices, and lifestyle were not controlled in this study and should be considered in future research.

Metronidazole remains the first-line antimicrobial therapy for bacterial vaginosis (BV); however, its clinical utility is significantly limited by high recurrence rates of 30–70% within 12 months post-treatment. These recurrences are multifactorial, attributed to antibiotic-induced disruption of the indigenous vaginal Lactobacillus-dominant microbiota, potential sexual transmission dynamics, and the persistence of resilient polymicrobial biofilms formed by BV-associated bacteria (e.g., Gardnerella vaginalis, Atopobium vaginae) that confer treatment resistance [20]. Concomitantly, metronidazole administration carries notable adverse effects that may compromise treatment adherence and tolerability. Gastrointestinal disturbances are prevalent, with nausea (reported in 20–30% of patients), vomiting (5–10%), and diarrhea (5–10%) occurring dose-dependently. Additionally, dysgeusia (a persistent metallic taste, affecting 10–25% of users) and oral complications such as black hairy tongue (a benign but distressing hyperpigmentation and elongation of filiform papillae, incidence <1%) are well-documented. These side effects arise from metronidazole’s systemic absorption and disruption of oral/gut microbiota, potentially leading to premature treatment discontinuation and subtherapeutic exposure—further exacerbating recurrence risks. While adjunctive probiotic therapy (e.g., oral or vaginal Lactobacillus strains) has been investigated to restore vaginal eubiosis and reduce recurrence rates by competitively excluding pathobionts and modulating biofilm integrity, current evidence does not support dose reduction of metronidazole when combined with probiotics. Clinical trials demonstrate that probiotics may modestly lower recurrence rates (by 10–15% in some cohorts) but do not enhance the immediate bactericidal efficacy of metronidazole against the dense biofilm-embedded BV consortia [21]. Crucially, subtherapeutic metronidazole dosing—whether through intentional reduction or poor adherence due to side effects—risks inadequate eradication of anaerobic pathogens, potentially selecting for resistant strains and accelerating recurrence. Thus, standard-dose metronidazole (500 mg twice daily orally for 7 days or 0.75% vaginal gel once daily for 5 days) remains imperative for initial cure, with probiotics considered only as a supplemental strategy post-antibiotic therapy to consolidate microbiota recovery, not as a dose-sparing intervention. Rigorous dose de-escalation studies with robust pharmacokinetic/pharmacodynamic modeling are lacking, and any deviation from established regimens is not evidence-based and may compromise therapeutic outcomes [5], [16].

Probiotics are live microorganisms that confer health benefits when consumed in adequate quantities. They have been shown to be effective in preventing and treating a variety of gastrointestinal infections, and there is growing interest in their potential for use in vaginal infections [22]. Probiotics have proven to be an efficient preventative measure for recurrent BV following treatment irrespective of the administration method. Many studies have shown an improvement in BV cure rate with probiotic supplementation (i.e. [23]). Nevertheless, comprehensive research on probiotic supplementation as a preventive measure for BV recurrences is lacking [19]. A systematic review have shown the effectiveness of probiotics in preventing BV recurrences within intervals of 1 to 3 months [10]. A prior narrative review has also indicated the potential of probiotics as a prophylactic agent against BV relapses. However, the systematic review had limitations regarding the number of searched databases, inclusion of small sample sizes, and absence of a standardized operational definition for BV recurrence and clarity in reporting [24]. Additionally, while Udjianto et al., have reported positive findings, a rcent systemativ review suggest that probiotics have no impact on increasing BV cure rates, lack strong positive effects, and have minimal significant effects when added to antibiotic regimens [14]. These disparities in research results can be associated with variations in investigation design, such as differences in the ethnicities of the research samples, the probiotics’ species and dosage, and how each strain modulates the vaginal microbiome. For example, past studies have highlighted that different strains of Lactobacillus have distinct levels of lactic acid production. Furthermore, women from diverse ethnicities exhibit various vaginal pH levels. The results indicate that the effectiveness of probiotics in treating BV may differ across diverse populations [25]. Other factors such as dietary habits, hygiene practices, and lifestyle were not controlled in this study and should be considered in future research.

The main aim of this clinical trial was to evaluate the efficacy of probiotics as an additional therapy to metronidazole in the management of BV. The findings of this investigation demonstrate that the co-administration of probiotics and metronidazole has promising outcomes. The individuals subjected to this combined therapy manifested greater alleviation of symptoms, implying that probiotics may have advantageous effects in the treatment of BV. This finding is in line with existing research showing the potential of probiotics in restoring balance to the vaginal microbiome.

The study also demonstrated a significant decrease in BV recurrence among the group receiving probiotics. This is a common and distressing issue for patients. The findings of the research propose that probiotics may aid in the prevention or postponement of recurrences, thus enabling a substantial benefit in the condition’s long-term management.

The study on the influence of probiotics on the vaginal microbiome offers valuable knowledge about the underlying processes. The research observed modifications in the composition of the vaginal microbiota, which implies that probiotics may enhance the propagation of beneficial *Lactobacillus* spp. This is vital as the dominance of Lactobacillus is linked with vaginal wellbeing and reduced vulnerability to infections [26]. While the positive clinical outcomes are noteworthy, it is crucial to take into account the viewpoints and quality of life of the patients. Conducting qualitative evaluations, including patient-reported outcomes and surveys, may offer a more comprehensive comprehension of the effect of this therapy on the everyday lives of women who are experiencing BV [27]. Many investigations employ antibiotic intervention, such as the administration of 500 mg of oral metronidazole twice daily for a duration of 7 days, in conjunction with simultaneous probiotic therapy, as a means to enhance the efficacy of treatment and mitigate the risk of BV relapse [28], [29]. However, in this study, we administered probiotics at intervals (once every three nights) after a course of antibacterial treatment to maintain the vaginal bacterial flora for a longer period during a menstrual cycle, in an attempt to achieve better therapeutic effects.

Nugent’s score and Amsel’s criteria are two commonly used methods for the diagnosis of bacterial vaginosis (BV). Nugent’s score is a Gram stain scoring system that evaluates the presence of bacterial morphotypes, including large Gram-positive rods (*Lactobacillus* morphotypes), small Gram-variable rods (*Gardnerella vaginalis* morphotypes), and curved Gram-variable rods (*Mobiluncus* spp. morphotypes). A score of 7 to 10 is consistent with BV. On the other hand, Amsel’s criteria involve clinical parameters such as the presence of thin, white, yellow, homogeneous discharge, elevated vaginal pH (>4.5), the presence of more than 20% clue cells on microscopic examination, and a positive whiff test [30], [31]. A study found that the Amsel criteria have been validated as equivalent to Nugent scoring when diagnosing BV, and a combination of Amsel’s criteria and Nugent scoring may be beneficial for an accurate diagnosis of BV due to an assessment on both clinical and laboratory grounds [32]. However, the sensitivity and specificity of Amsel’s criteria have been reported to range from 37% to 70% and 94% to 99%, respectively, with moderate reproducibility [33]. In comparison, Nugent’s score is considered the gold standard for diagnosing BV, with a score of 7 to 10 being consistent with BV without culture. It is important to note that Nugent scoring is time-consuming and requires the expertise of a trained microscopist [34]. However, the Amsel criteria have limitations in sensitivity and specificity. A combination of Amsel and Nugent scoring could provide more accurate diagnoses, reducing false positives and false negatives.

A study comparing Amsel’s criteria with low and high Nugent’s scores found that Amsel’s criteria showed a sensitivity of 100% with high Nugent’s scores and 81% with low scores, indicating that Amsel’s criteria may be more sensitive in cases of high Nugent’s scores [35]. Another study found that Amsel's criteria without utilizing staining methods could be misleading, with a high specificity but a significant number of false positives [36], [37].

## Limitations

The sample size was comparatively small, and the research concentrated solely on Iranian women. Expanding the research to a more diverse population and a larger cohort would improve findings’ generalisability. Moreover, longer-term follow-up could offer insights into the durability of effects. 

## Conclusion

The study introduces several promising future research paths. Investigating the most suitable probiotic strains, dosages, and treatment durations for bacterial vaginitis could help to refine treatment protocols. Additionally, investigating the possibility of tailored microbiome-focused treatments for BV presents considerable prospects for enhancing results. 

A number of clinical trials have investigated the use of probiotics as adjunctive therapy to metronidazole in the treatment of BV. No all have shown promising results. As a well-designed clinical trial the current study is an another building block for the use of probiotics to support the therapy for BV. However, further studies are needed to confirm these findings and to determine the optimal probiotic strains and dosage for the treatment of BV. 

## Notes

### Authors’ ORCIDs

- Vahedpoor Z: https://orcid.org/0000-0002-3041-9908
- Azadchehr MJ: https://orcid.org/0000-0001-6877-8896
- Nazeri M: https://orcid.org/0000-0001-7060-5011

### Ethical approval 

The study protocol was approved by the Research Ethics Committee of KAUMS, Iran. Before enrolment, all patients provided written informed consent in accordance with the tenets of the Declaration of Helsinki, after which vaginal specimens were collected.

### Funding

The research has been financially supported by Kashan University of Medical Sciences (98088) of research deputy of Kashan University of Medical Sciences and the participants are appreciated.

### Acknowledgments

This research is a part of the M.D. thesis. The authors would like to thank the Clinical Reasearch Development Unit of Kashan Sahid Behshti hospital.

### Competing interests

The authors declare that they have no competing interests.

## Figures and Tables

**Table 1:**
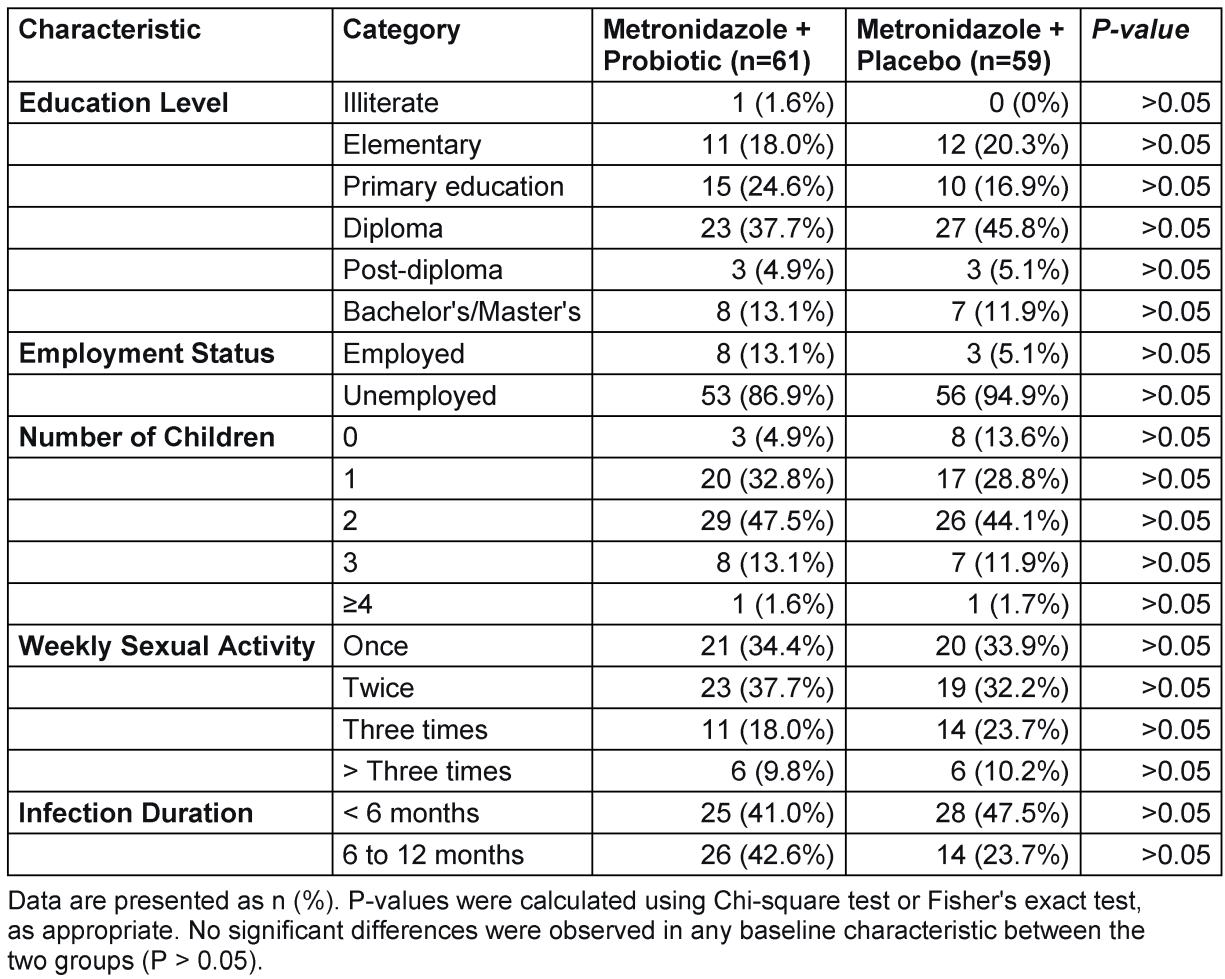
Descriptive characteristics of patients in the two groups

**Table 2:**
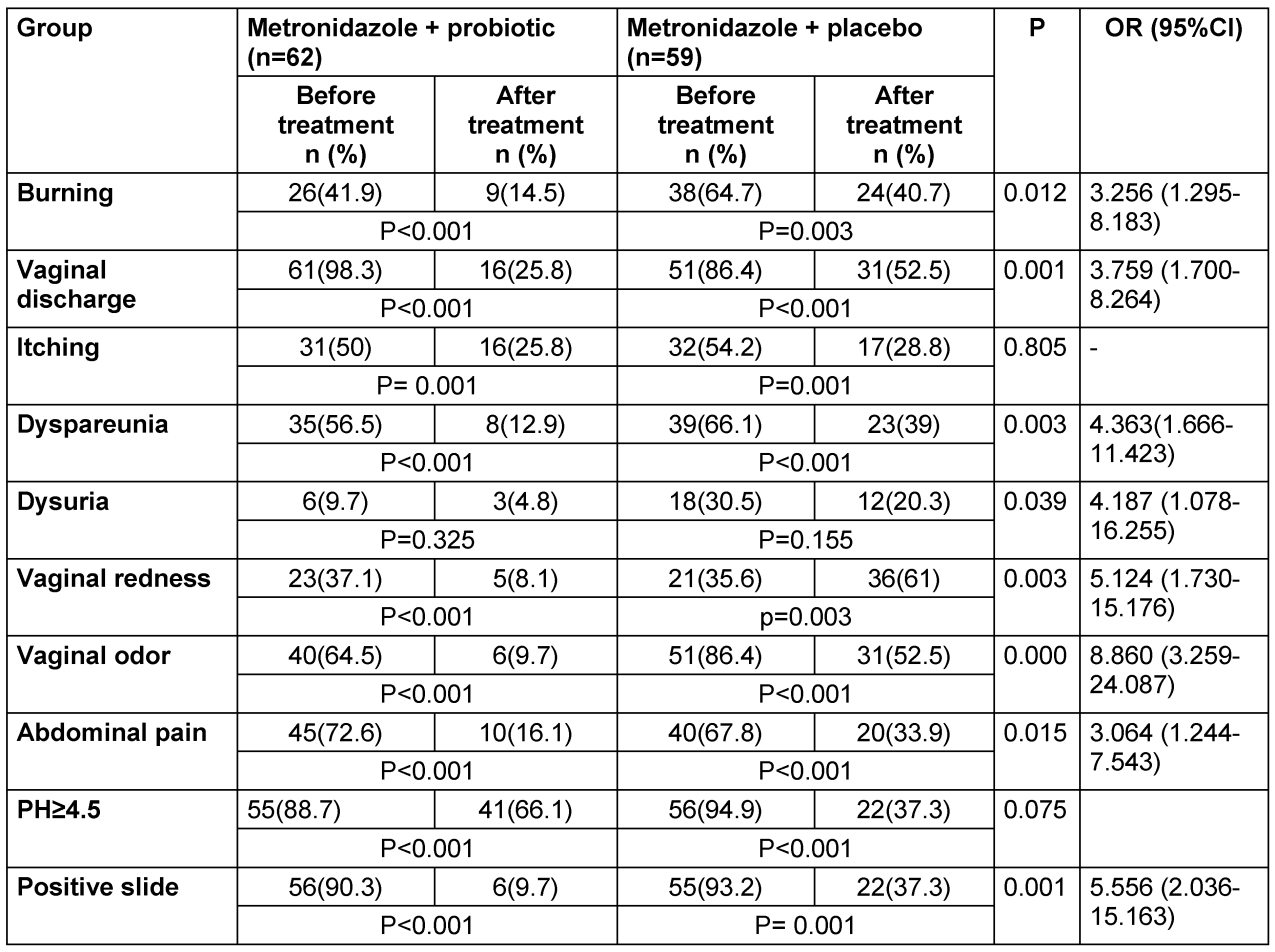
Comparison of the desired variables in two intervention and control groups

**Table 3:**
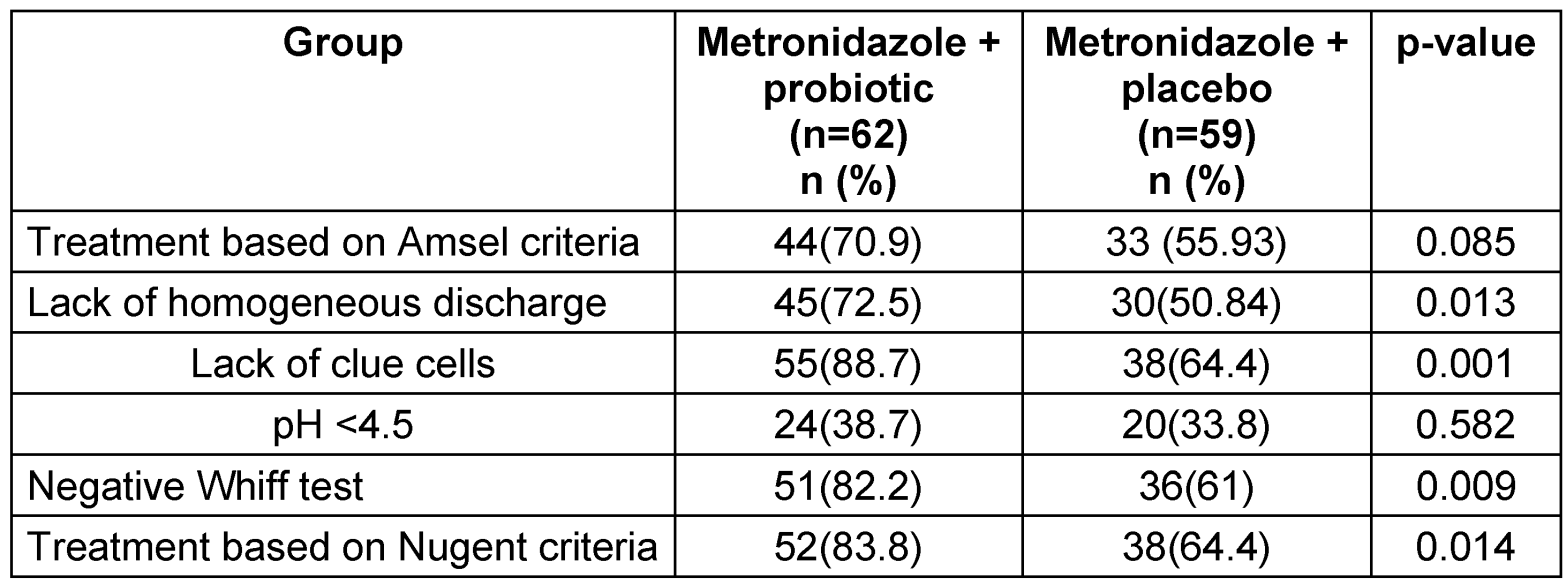
Frequency distribution of the desired variables in terms of recovery after intervention in two groups
